# A Mediation Analysis of Trauma Symptoms on the Well-Being in Caregivers of Children with Medical Complexity

**DOI:** 10.3390/healthcare13182332

**Published:** 2025-09-17

**Authors:** Courtney M. Holmes, Kanako Iwanaga, Tiffany Kimbrough, Simran Singh, Marcia A. Winter, Ayomide Popoola, Genevive Heymann, Makayla Burton, Heather A. Jones

**Affiliations:** 1 Department of Rehabilitation Counseling, Virginia Commonwealth University, Richmond, VA 23298, USA; iwanagak@vcu.edu (K.I.); singhs62@vcu.edu (S.S.); 2 Department of Pediatrics, Virginia Commonwealth University Health, Richmond, VA 23298, USA; tiffany.kimbrough@vcuhealth.org; 3 Department of Psychology, Virginia Commonwealth University, Richmond, VA 23298, USA; mawinter@vcu.edu (M.A.W.); popoolaaa@vcu.edu (A.P.); hjones7@vcu.edu (H.A.J.); 4 College of Humanities and Sciences, Virginia Commonwealth University, Richmond, VA 23284, USA; heymannj@vcu.edu (G.H.); burtonmr@vcu.edu (M.B.)

**Keywords:** medical complexity, parent mental health, trauma, resilience

## Abstract

**Background/Objectives**: Parent caregivers of children with medical complexity experience high levels of chronic stress and trauma symptoms. Despite ongoing and continuous involvement with the healthcare system, parents often do not seek support for their own mental health concerns. Parent mental wellness is a critical component of reducing family distress. This study aimed to explore how various factors such as resilience, coping, and support impacted well-being and trauma symptom severity. **Methods**: Parents/caregivers from a complex care clinic in a mid-Atlantic children’s hospital were recruited for this study. A total of 125 participants completed the study which included a battery of measures focused on trauma symptoms, well-being, coping, and resilience. **Results**: PTSD symptom severity was significantly negatively related to resilience and subjective well-being. Significant mediators included informational support, indicating that collaboration between healthcare providers and caregivers/parents is critical. **Conclusions**: This study highlights potential target intervention areas to promote well-being and reduce trauma symptom severity, such as resiliency promotion and enhancement and increasing informational support provided by healthcare professionals.

## 1. Introduction

Parents and caregivers of children with medical complexity (CMC) face untenable levels of stress [1,2,3,4] starting with the initial diagnosis, continuing with unpredictable health outcomes with intermittent health crises. For some families, this starts with pre-term birth, compounded with stays in the neonatal intensive care unit (NICU). It is well documented that pre-term birth, NICU treatment, and receiving a diagnosis requiring ongoing complex medical care are correlated with negative and critical mental health outcomes such as post-traumatic stress, anxiety, and depression [5,6]. Parent caregivers consistently report elevated levels of depression, anxiety, fatigue, distress, poor physical health, cardiovascular risks, headaches, decreased cognitive function, isolation, emotional distress, and other mental health complaints [2,7,8,9]. Parents are likely to experience medical trauma during their children’s lives, putting them at a higher risk for developing Post-Trauma Stress Disorder (PTSD), depression, anxiety, and complicated grief [10,11]. Caregiver burden and strain are well documented in parents of CMC [3,12] as negative impacts include physical, psychological, and financial decline [3,13]. This occurs as parents are now required to manage complex care plans which involve specialized medical tasks, coordinating with multiple providers, and navigating medical technology, amidst household and financial responsibilities [4,14]. Moreover, family members report that despite ongoing interfacing with the healthcare system, mental and behavioral healthcare needs remain unmet [7,15,16]. More specifically, fragmented healthcare systems, insufficient and untrained home-based services, and complex medical decisions hinder appropriate care for CMC, thereby exacerbating the distress [1,17]. Such experiences can lead to unhealthy coping mechanisms and various negative health outcomes, including heightened anxiety and depression, increased substance use, cardiovascular problems, chronic pain, and other physical illnesses [18].

Studies have shown the benefits of care coordination and patient-centered care for families with CMC. In NICU studies, healthcare workers attuned to supporting mothers’ needs while promoting collaborative decision-making during labor and delivery contribute positively to childbirth experiences [19,20]. Labor and delivery workers’ emphasis on informed consent, effective communication, and consideration of women’s values and preferences during labor can enhance the psychological health and well-being of both the mother and the family unit [19,20]. Alternatively, a reduced sense of control for parents, invasive medical efforts with minimal communication of rationale, and lack of sufficient support from partners and healthcare workers can result in traumatic birth experiences [21,22]. Post-NICU, studies have indicated significant benefits of effective care coordination to reducing burden for families [4,23]. Despite the documented importance of collaboration between healthcare professionals and parent caregivers, parents of CMC often report feeling devalued and unheard in hospital setting [24]. Informational support has also been linked to resilience in parents of CMC [25] and collaboration and trust with medical providers has been shown to potentially reduce symptoms of medical trauma [26]. Considering the rate of re-admissions of CMC (e.g., two-thirds of re-admission within 90 days) [27], the importance of examining trauma and well-being in this population cannot be understated. The promotion of resilience and well-being to ensure optimal health and functioning of CMC families. Resilience, the capacity to heal from negative events, can be developed in individuals and families with support and intervention.

This study aimed to further explore how various factors such as resilience, coping, and support impacted well-being and trauma symptom severity in parents of CMC as further understanding could support interventions designed to reduce the impact of trauma and improve well-being and family functioning [26,28]. Specifically, we examined the direct and indirect associations between PTSD symptoms and subjective well-being (SWB) via the proposed mediators. It was hypothesized that the relationship between PTSD symptom severity and SWB would be mediated by resilience, emotional support, informational support, and adaptive coping.

## 2. Materials and Methods

### 2.1. Procedure

This study was approved by the Institutional Review Board of [university name withheld for review] (HM20030584, approved 14 August 2024). Upon approval, all enrolled parents and caregivers of CMC in a complex care clinic at a MidAtlantic children’s hospital were recruited via email. Parents/caregivers were also recruited in person during clinic appointments by a research assistant. This study was funded by an internal university grant, and participants were offered a $30 gift card for the completion of the survey. Inclusion criteria included: age of 18+ of the parent and child enrolled in the complex care clinic, and English speaking. Consenting participants completed a battery of measures with a completion time of less than 20 min, and all surveys were collected with REDCap [29,30]. A total of 250 parents and caregivers were invited to participate in the survey, and 125 completed it. Following data cleaning procedures, including addressing missing data, identifying outliers, and evaluating multicollinearity (see Section 2.4), a final sample of 121 participants was included in both the descriptive analyses and parallel mediation analysis.

### 2.2. Participants

The study includes 121 participants who are caregivers of children with medically complex needs. Participant demographic characteristics are presented in Table 1. The majority identified as White (*n* = 66, 52.8%) or Black/African American (*n* = 47, 37.6%) and female (*n* = 114, 91.2%), with a smaller proportion identifying as male (*n* = 9, 7.2%) or non-binary (*n* = 1, 0.8%). Annual household income levels varied: 24.8% (*n* = 31) reported incomes less than $25,000, 20.0% (*n* = 25) earned between $25,000 and $49,999; and 16.8% (*n* = 21) reported household incomes of $150,000 or more. In terms of marital status, 54.4% (*n* = 68) were married or partnered, 33.6% (*n* = 42) were single and had never been married or were not living with a romantic partner, and 8.8% (*n* = 11) were divorced or separated. In terms of family size, 35.2% (*n* = 44) of participants had one child, 34.4% (*n* = 43) had two children, and 17.6% (*n* = 22) had three children. A significant majority (92.0%, *n* = 115) reported having just one child with complex medical needs. Additionally, 75.2% (*n* = 94) indicated that their child spent time in the NICU after birth. Regarding health insurance, nearly half of the participants (49.6%, *n* = 62) reported using Medicaid or CHIP Medicaid, followed by 37.6% (*n* = 47) who reported having private insurance.

### 2.3. Measures

#### 2.3.1. Subjective Well-Being

Subjective well-being (SWB) was measured using the Satisfaction with Life Scale (SWLS) [31]. The SWLS is a 5-item scale designed to measure global cognitive judgments of one’s SWB (e.g., “in most ways my life is close to my ideal”). Each item is rated on a 7-point Likert scale (1 = *Strongly disagree* to 7 = *Strongly agree*), and scores were averaged to produce a final score ranging from 1 to 7. Higher scores indicate greater SWB. Diener and colleagues (1985) reported Cronbach’s alpha of 0.87 and a test–retest reliability of 0.82 for SWLS. In the present study, the Cronbach’s alpha was 0.87.

#### 2.3.2. PTSD Symptom Severity

The Structured Trauma-Related Experiences & Symptoms Screener (STRESS) [32] is a 10 to 15 min self-report instrument that assesses (1) lifetime exposure to several domains of potentially traumatic experiences in childhood and adulthood (52 items), (2) PTSD symptoms that map onto DSM-5 symptom criteria and applicable to one or more traumatic events (25 items), and functional impairment (6 items). For a total score, we summed only the 25 items related to symptom severity, from “stress1_b1” to “stress25_ds2,” to create the total score. Each item is rated on a 4-point Likert scale (0 = *None*, 1 = 1 *Day*, 2 = 2–3 *Days*, 3 = *Most Days*), with total scores ranging from 0 to 75. Higher scores indicate greater PTSD symptoms. For our sample, Cronbach’s alpha = 0.96. The STRESS has been tested in various adult populations with excellent internal consistency for total symptom severity (Cronbach’s alpha > 0.90), construct validity as indicated by a strong fit with the DSM-5 four-factor symptom structure, and concurrent validity when examined alongside other validated measures of internalizing and externalizing behavior problems. Further, total count of endorsed trauma types has been shown to associate with other validated measures of stress/trauma exposure and impairment [32,33].

#### 2.3.3. Resilience

The Brief Resilience Scale (BRS) [34] was used to assess resilience or the ability to bounce back from adverse events. It is composed of three positive valence items and three negative valence items [e.g., “I tend to bounce back quickly after hard times” (positive) and “I have a hard time making it through stressful events” (negative)]. Each item was rated on a 5-point Likert-type scale ranging from 1 (*strongly disagree*) to 5 (*strongly agree*), and negatively worded items were reverse-scored. Final scores were calculated by averaging the six items, resulting in a possible range of 1 to 5, with higher scores indicating greater resilience. The Cronbach’s alpha coefficients for the BRS were reported from 0.80 to 0.91, with test–retest reliability reported to range from 0.61 to 0.69 [35]. In the present study, the Cronbach’s alpha = 0.85.

#### 2.3.4. Emotional Support

Emotional Support was measured using the PROMIS Short Form v2.0—Emotional Support 4a [36]. The PROMIS Emotional Support 4a is a 4-item scale designed to assess perceived feelings of being cared for and valued as a person, and having confidant relationships (e.g., “I have someone who will listen to me when I need to talk”). Each item is rated on a 5-point Likert scale (1 = *Never* to 5 = *Always*), and scores were averaged to produce a final score ranging from 1 to 5, with higher scores representing greater emotional support. The manual reported strong internal consistency reliability for the PROMIS Emotional Support instruments, with Cronbach’s alpha typically above 0.90 in adult samples [36]. In the present study, the Cronbach’s alpha was 0.94.

#### 2.3.5. Informational Support

Informational Support was measured using the PROMIS Short Form v2.0—Informational Support 4a [37]. The PROMIS Informational Support 4a is a 4-item scale designed to assess the perceived availability of helpful information or advice (e.g., “I have someone to give me good advice about a crisis if I need it.”). Each item is rated on a 5-point Likert scale (1 = *Never* to 5 = *Always*), and scores were averaged to produce a final score ranging from 1 to 5, with higher scores indicating greater informational support. According to the manual [37], the PROMIS Informational Support instruments demonstrated high internal consistency, with Cronbach’s alpha values generally exceeding 0.90 in adult populations. In the present study, the Cronbach’s alpha = 0.94.

#### 2.3.6. Adaptive Coping

Adaptive Coping was measured using the Adaptive Coping subscale of the Coping Flexibility Scale [38]. The Adaptive Coping subscale is a 5-item measure designed to assess the ability to generate and implement alternative coping strategies when existing coping efforts are ineffective (e.g., “when a stressful situation has not improved, I try to think of other ways to cope with it”). Each item is rated on a 4-point Likert scale (1 = *Not applicable* to 4 = *Very applicable*), and scores were averaged to produce a final score ranging from 1 to 4, with higher scores indicating greater adaptive coping flexibility. The authors reported Cronbach’s alpha coefficients of 0.86 and 0.88 for the Adaptive Coping subscale across samples of Japanese college students and employees [38]. For our sample, the Cronbach’s alpha = 0.86.

### 2.4. Data Analysis

All statistical analyses were conducted using IBM SPSS Statistics (Version 29.0). Descriptive statistics were computed to obtain the means and standard deviations for all independent and dependent variables. Correlation analysis was used to examine the relationships among variables. A parallel mediation analysis was conducted using the PROCESS macro for SPSS (Version 4.0) [39] to estimate the total, direct, and indirect effects of PTSD symptom severity on SWB through the proposed mediators: resilience, emotional support, informational support, and adaptive coping. This analysis used a bootstrapping approach with 5000 samples [40,41].

A total of 125 participants completed the survey. However, some participants had missing responses on one or more scales. Cases with more than 50% missing items on any scale were excluded from the analysis. For cases with less than 50% missing data on a given scale, missing values were replaced using mean imputation based on the available items within that scale. Because only a small amount of data was missing and there was no clear pattern, we assumed the data were Missing at Random (MAR), which made mean imputation appropriate. Approximately 1 to 3 cases were recovered per variable through this method. As a result, the number of valid cases slightly varied across measures (e.g., between 123 and 125). For the descriptive and parallel mediation analysis, only participants with complete data on all study variables were included, resulting in a final analytic sample of 121. Multivariate outliers were assessed using Mahalanobis distance, and none were identified. Multicollinearity was evaluated using variance inflation factors (VIFs) and tolerance values. All VIF values were below 4.0, and all tolerance values exceeded 0.20, indicating no issues with multicollinearity.

## 3. Results

### 3.1. Descriptive Statistics and Correlations

Means, standard deviations, and the correlation matrix for variables in the hypothesized mediational model are shown in Table 2. The mean score for PTSD symptom severity was 26.76 (SD = 18.35). The mean score for SWB was 4.12 (SD = 1.39), while the mean score for resilience was 3.30 (SD = 0.71). For perceived social support, the mean score for emotional support was 3.63 (SD = 1.08) and for informational support was 3.58 (SD = 1.05). The mean score for adaptive coping was 2.77 (SD = 0.68).

Results from the correlational analysis indicated that PTSD symptom severity was significantly negatively associated with SWB (r = −0.33, *p* < 0.01), resilience (r = −0.22, *p* < 0.05), emotional support (r = −0.34, *p* < 0.01), and informational support (r = −0.36, *p* < 0.01). No significant association was found between PTSD symptom severity and adaptive coping (r = −0.05, n.s.). SWB was positively correlated with resilience (r = 0.38, *p* < 0.01), emotional support (r = 0.34, *p* < 0.01), informational support (r = 0.42, *p* < 0.01), and adaptive coping (r = 0.21, *p* < 0.05). Resilience showed significant positive correlations with emotional support (r = 0.25, *p* < 0.01), informational support (r = 0.30, *p* < 0.01), and adaptive coping (r = 0.28, *p* < 0.01). Moreover, emotional support and informational support were highly correlated (r = 0.84, *p* < 0.01), and both were positively associated with adaptive coping (r = 0.22, *p* < 0.05; r = 0.24, *p* < 0.01, respectively).

### 3.2. Parallel Mediation Analysis

**Figure 1:**
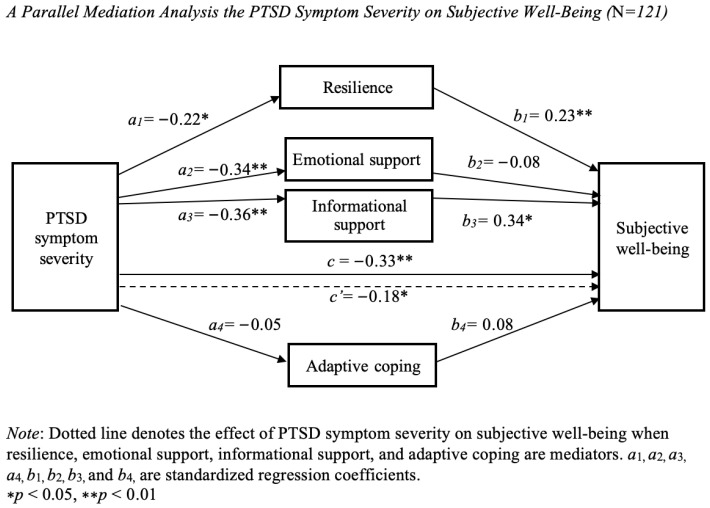
The parallel mediation model accounted for 27.92% of the variance in SWB, R = 0.53, R^2^ = 0.28, F (5, 115) = 8.91, *p* < 0.001, which is a large effect size [42].

A parallel mediation analysis was computed to evaluate resilience, emotional support, informational support, and adaptive coping as mediators (Ms) in the relationship between PTSD symptom severity (X) and SWB (Y). A graphical representation of this model and information on the standardized path coefficients is presented in Figure 1.

#### 3.2.1. Total Effect

As can be observed in Figure 1, PTSD symptom severity was negatively linked to SWB [c = −0.33, *p* < 0.001, 95% confidence interval (CI; −0.50, −0.16)].

#### 3.2.2. Direct Effects

The relationship between PTSD symptom severity and each of the mediators was assessed. PTSD symptom severity was directly, negatively related to resilience [a_1_ = −0.22, *p* < 0.05, 95% CI (−0.39, −0.04)], emotional support [a_2_ = −0.34, *p* < 0.001, 95% CI (−0.51, −0.17)], and informational support [a_3_ = −0.36, *p* < 0.001, 95% CI (−0.52, −0.19)]. On the other hand, PTSD symptom severity had no direct effect on adaptive coping [a_4_ = −0.05, *p* = 0.56, 95% CI (−0.23, 0.13)]. The relationship between each of the mediators and SWB, controlling for PTSD symptom severity, was assessed. Resilience [b_1_ = 0.23, *p* < 0.01, 95% CI (0.06, 0.40)] and informational support [b_3_ = 0.34, *p* < 0.05, 95% CI (0.04, 0.63)], were significantly positively associated with SWB after controlling for PTSD symptom severity and other mediators. In contrast, emotional support [b_2_ = −0.08, *p* = 0.58, 95% CI (−0.37, 0.21)], and adaptive coping [b_4_ = 0.08, *p* = 0.37, 95% CI (−0.09, 0.24)] were not significantly associated with SWB. Importantly, the direct effect of PTSD symptoms severity on SWB was reduced but remained statistically significant after accounting for the mediators (c′ = −0.18, *p* < 0.05, 95% CI (−0.35, −0.02), suggesting that the relationship was partially mediated by resilience and informational support.

#### 3.2.3. Indirect Effects

The mediation was tested by estimating the indirect effects of PTSD symptoms severity on SWB through resilience, emotional support, informational support, and adaptive coping. Specific indirect effects were considered statistically significant if the bias-corrected bootstrap confidence intervals (CIs) for the products of these paths did not include zero [39]. Bootstrapping with 5000 samples revealed significant indirect effects of PTSD symptom severity on SWB through resilience (point estimate = −0.05, [95% CI: −0.11, −0.01]), and through the informational support (point estimate = −0.12, [95% CI: −0.25, −0.02]), but not through emotional support (point estimate = 0.03, [95% CI: −0.08, 0.11]), or adaptive coping (point estimate = −0.12, [95% CI: −0.03, 0.02]).

## 4. Discussion

This study examined how resilience, perceived support, and adaptive coping mediate the relationship between PTSD symptom severity and subjective well-being in caregivers of CMC. The findings revealed that informational support and resilience significantly mediated this relationship, suggesting that caregivers who report higher levels of these protective factors tend to experience greater well-being despite the presence of trauma symptoms. These mediating mechanisms represent modifiable targets for intervention, especially critical for caregivers who often face high levels of stress and uncertainty. Informational support refers to access to useful, accurate, and timely information that helps caregivers manage care-related stressors, such as learning facts about their child’s condition, receiving guidance from trusted professionals, or connecting with others who have navigated similar challenges [36]. For families with CMC, this form of support can be understood through the lens of collaborative, team-based care. When caregivers are actively involved in their child’s care and feel supported with knowledge, tools, and professional guidance, their sense of well-being is likely to improve.

In practice, informational support may be offered through structured communication tools like care binders, electronic portals, and individualized health education resources [43,44]. Parent education sessions led by multidisciplinary teams can also help caregivers build confidence in their ability to care for their child. On a system level, integrated care models such as the patient-centered medical home facilitate coordinated, family-centered communication between healthcare professionals and families [45] and represent a feasible intervention for underserved, diverse families [46]. Other scalable models include care coordination teams, family navigators, or peer mentorship programs, which can be particularly helpful during transitions like NICU discharge or surgery recovery [47,48]. Because informational support involves concrete tools and guidance, it may be more scalable and sustainable than interventions aimed at changing emotional or clinical states. Prior studies have emphasized the critical role of this type of support in complex care settings [12,49]. This study supports qualitative analysis suggesting that informational support was connected to personal resilience [25]. This offers a practical entry point for support services, especially for community organizations or care coordinators.

Although both informational and emotional support were positively correlated with well-being, only informational support significantly mediated the relationship between PTSD symptoms severity and subjective well-being in this study. While emotional support includes empathy, compassion, and feeling understood by others, informational support provides more concrete, action-oriented guidance that may be especially helpful when caregivers are trying to make decisions, plan for care, or cope with crises. These results may indicate that the ability to access and apply helpful information is more immediately protective in the context of caregiving stress. The difference may also be explained by theoretical frameworks. According to Lazarus and Folkman’s (1984) transactional model of stress and coping, informational support aligns more closely with problem-focused coping, helping individuals assess, plan, and take action, whereas emotional support is aligned with emotion-focused coping, which may be more beneficial when stressors are less controllable [50]. Informational support may also improve a caregiver’s self-efficacy, or confidence in their ability to manage challenges, which has been associated with better adjustment and lower distress in previous studies on parents of children with chronic conditions [51,52]. These overlapping mechanisms may help explain why informational support, not emotional support, emerged as a significant mediator in the pathway from trauma symptoms to well-being.

When conducting care coordination plans, providers must also consider the significant role of factors such as cultural background, language preferences, perception of health outcomes, and the method of communicating information. Consideration of cultural values has been found to be important in relation to information needs, level of honesty in communication, and concordance between patient and provider values [53,54,55,56]. Previous researchers have highlighted the importance of altering health-related communication based on cultural factors as the congruence between cultural characteristics and communication can improve message effectiveness, understanding, and deeper processing [57]. Differences in health and treatment outcomes have been found between individualistic and collectivistic cultures based on promotion vs. prevention approaches [57], communicating with the individual or the family system [54], and emphasis on social values and norms [58]. Therefore, considerations regarding cultural differences are crucial to ensure optimal effectiveness of the support provided.

In addition to informational support, resilience significantly mediated the relationship between PTSD symptom severity and subjective well-being. This finding highlights the role of resilience as a protective factor that may help caregivers of CMC manage psychological stress. Often defined as the ability to adapt and recover from adversity, resilience can enable caregivers to reframe challenges, maintain motivation, and stay engaged in their caregiving roles, even when faced with trauma-related symptoms. While some aspects of resilience may be innate, it is widely understood as a quality that can be strengthened. Interventions such as cognitive-behavioral therapy, mindfulness, narrative-based approaches, and positive psychology practices have shown promise in enhancing resilience in parent and caregiver populations [59,60]. Ensuring access to these resources, particularly within the context of complex care, may help reduce barriers and provide caregivers with practical strategies to support their well-being. Public health policy and training initiatives can be informed by these data as access to mental health support can be promoted with healthcare policy initiatives, greater insurance coverage, and well-trained medical providers. Trauma-informed health systems are critical to more effectively supporting CMC families, as an informed workforce on all levels can reduce traumatization and support well-being.

Although emotional support and adaptive coping were positively correlated with well-being, neither significantly mediated the relationship between PTSD symptoms and well-being in this study. One possible explanation is the high degree of overlap between emotional and informational support, which may have introduced shared variance and reduced the unique effect of emotional support. Another interpretation is that certain forms of support may be more effective than others in managing trauma-related stress. While emotional support offers general comfort and validation, informational support and resilience may more directly influence caregivers’ perceptions of control and capacity to manage the demands of complex medical care. Similarly, the non-significant finding for adaptive coping may reflect variability in how caregivers interpret or apply coping strategies under conditions of chronic and prolonged stress.

Finally, the continued significance of the direct effect between PTSD symptom severity and subjective well-being suggests that other contributing factors were not captured in the current model. Recent studies point to a range of psychological and contextual influences that may also shape caregiver well-being. Caregiver burden has been consistently linked to emotional strain, with many caregivers reporting ongoing fatigue, role conflict, and reduced quality of life [61]. Social isolation, particularly in the post-pandemic context, has been associated with elevated anxiety and depression [62]. Financial stressors, such as out-of-pocket expenses and disrupted employment, remain common among families caring for children with complex needs [63]. Sleep difficulties are also prevalent and have been shown to contribute to heightened psychological distress [64], and limited access to respite care continues to limit caregivers’ opportunities for rest and self-care [65]. These factors may interact with trauma symptoms in complex and cumulative ways. Future research should incorporate these variables to gain a more comprehensive understanding of caregiver well-being and to inform interventions that address the broader context in which caregiving occurs.

### Limitations

This study has several limitations that should be considered when interpreting the findings. First, the cross-sectional design limits the ability to make causal inferences. Although mediation analysis was used to examine potential pathways, the data do not establish the temporal ordering of variables. Second, the single-informant data were based entirely on caregiver self-report, which may introduce response biases such as recall errors or the tendency to respond in socially desirable ways. This is particularly important when measuring sensitive constructs like PTSD symptoms and well-being. Third, the sample was drawn from a single tertiary care center, which may limit generalizability. The demographic composition of the sample may not reflect the broader population of caregivers of children with medical complexity, especially in terms of racial, ethnic, or socioeconomic diversity. Finally, while the study focused on several important protective and supportive factors, it is likely that other relevant variables, such as caregiver burden, sleep quality, financial stress, or access to respite and mental healthcare, also contribute to caregiver well-being. These factors were not captured in the present analysis and should be considered in future research.

## 5. Conclusions

Providers of families with CMC can benefit from the outcomes of this mediation analysis. Resilience and well-being can be fostered and improved through the delivery of collaborative and intentional informational support. Parents and caregivers of CMC often provide a significant amount of healthcare within the family context. When provided the opportunity to learn from and with healthcare providers, this can serve as a highly effective coping strategy to bolster well-being and foster resilience. This study serves to support the ongoing developments in collaborative, patient-centered care models for CMC and provides support for an increase in integrated care clinics that serve CMC and their families.

## Figures and Tables

**Table 1:** Participant Demographic Characteristics (*n* = 125).

	Frequency	Percent
Race		
Black or African American	47	37.6
White	66	52.8
American Indian or Alaska Native	1	0.8
Asian	2	1.6
Multiracial	6	4.8
Missing	3	2.4
Gender Identity		
Man	9	7.2
Woman	114	91.2
Non-Binary	1	0.8
Missing	1	0.8
Annual Household Income		
Less than $25,000	31	24.8
$25,000–$49,999	25	20
$50,000–$74,999	17	13.6
$75,000–$99,999	12	9.6
$100,000–$149,999	17	13.6
$150,000 or more	21	16.8
Missing	2	1.6
Marital Status		
Married or partnered	68	54.4
Divorced or separated	11	8.8
Widowed	1	0.8
Single, never been married-not living with romantic partner	42	33.6
Missing	3	2.4
# of Children		
1	44	35.2
2	43	34.4
3	22	17.6
4	5	4
5 or more	11	8.8
# of Children Who Have Complex Medical Needs		
1	115	92
2	7	5.6
3	2	1.6
4 or more	1	0.8
Did You Child Spend Time in the NICU After Birth?		
Yes	94	75.2
No	31	24.8
Main Insurance		
Medicaid or CHIP Medicaid	62	49.6
Medicare	6	4.8
other public insurance	5	4
private insurance	47	37.6
none/uninsured	3	2.4
missing	2	1.6

**Table 2:** Means, Standard Deviations, and Correlations Among Study Variables (*n* = 121).

Variables	1	2	3	4	5	6
1. PTSD symptom severity	—					
2. Subjective well-being	−0.33 **	—				
3. Resilience	−0.22 *	0.38 **	—			
4. Emotional support	−0.34 **	0.34 **	0.25 **	—		
5. Informational support	−0.36 **	0.42 **	0.30 **	0.84 **	—	
6. Adaptive coping	−0.05	0.21 *	0.28 **	0.22 *	0.24 **	—
Mean	26.76	4.12	3.30	3.63	3.58	2.77
SD (Possible score range)	18.35 (0–75)	1.39 (1–7)	0.71 (1–5)	1.08 (1–5)	1.05 (1–5)	0.68 (1–4)

* *p* < 0.05, ** *p* < 0.01.

## Data Availability

This manuscript conforms to MDPI’s policies as described. The datasets presented in this article are not readily available as they are protected through IRB approval. Requests to access the datasets should be directed to the corresponding author.

## Abbreviations

The following abbreviations are used in this manuscript:

CMC	Children with medical complexity
PTSD	Post traumatic stress disorder
NICU	neonatal intensive care unit
SWB	subjective well-being
